# Over-the-counter and compounded mouthwashes for radiotherapy-induced xerostomia: a randomized controlled trial

**DOI:** 10.1590/1807-3107bor-2025.vol39.057

**Published:** 2025-06-02

**Authors:** Isabella Silveira SAVO, Samanta Vicente de OLIVEIRA, Gabriela Banacu de MELO, Celso LEMOS, Camila de Barros GALLO

**Affiliations:** (a) Universidade de São Paulo – School of Dentistry, Department of Stomatology, São Paulo, SP, Brazil.

**Keywords:** Xerostomia, Radiotherapy, Saliva, Artificial, Mouthwashes, Quality of Life

## Abstract

Head and neck radiotherapy quantitatively and qualitatively compromises salivary flow, and salivary substitutes have the potential to alleviate the symptoms. This study aims to assess the effectiveness of two salivary substitutes in relieving symptoms of radiotherapy-induced xerostomia through a double-blind randomized controlled trial. Twenty-four patients were selected from a dental oncology clinic and randomly assigned to the over-the-counter or compounded mouthwash group. Each patient was instructed to rinse their oral cavity with the assigned solution three times a day for 30 days. Both participants and researchers were blinded to the product used during the trial. The impact of xerostomia was assessed using a numerical rating scale and validated questionnaires on oral health-related quality of life and xerostomia, administered before and after the intervention and subsequently compared. Both groups exhibited a statistically significant reduction in xerostomia symptoms. The studied salivary substitutes produced transient beneficial effects on complaints of radiotherapy-induced xerostomia.

## Introduction

Xerostomia is the subjective sensation of dry mouth and may be associated with salivary gland hypofunction, and consequently, reduced salivary secretion. Clinically, this combination is characterized by thick and frothy saliva, dry oral mucosa, fissured tongue dorsum, and atrophy of filiform papillae. Several factors contribute to the decrease in salivary flow, including systemic diseases such as Sjögren’s syndrome, diabetes, HIV, and hepatitis C; medications; habits like smoking and mouth breathing; as well as chemotherapy and radiotherapy for head and neck cancer.^1^

Patients exposed to high doses of ionizing radiation over extensive head and neck fields experience a significant functional impact because of reduced salivary secretion. Saliva plays a crucial role in protecting the oral mucosa and tooth structure through its lubricating, buffering, and antimicrobial properties. The decrease in salivary flow exacerbates other complications associated with head and neck radiotherapy, such as dysgeusia, dysphagia, opportunistic fungal infections, and radiation-associated caries. It also impairs other oral functions such as speaking, chewing, and swallowing, which can hinder patients’ ability to eat, potentially compromising their nutritional status and overall quality of life.^1-5^

Currently, there are several therapeutic strategies for managing hyposalivation. Interventions aimed at increasing salivary flow are generally preferred and include parasympathomimetic agents (pilocarpine, cevimeline, and bethanechol) or cytoprotective drugs (amifostine and palifermin). The effectiveness of these substances depends on the presence of some residual gland function and on the absence of other comorbidities or intolerable adverse effects that might limit their benefit or indication. The timing of the intervention is also crucial; a preventive approach, whether pharmacological or alternative (photobiomodulation), tends to be more effective in controlling radiation-induced xerostomia. Therefore, selecting the most appropriate therapy requires careful consideration of several factors to achieve the best possible cost-effectiveness. In this context, salivary substitutes may offer a safe, low-cost, and accessible option with minimal adverse effects, helping alleviate the symptoms of radiation-induced xerostomia.^1,5,6^

Salivary substitutes contain substances that mimic the action of mucin, such as oxygenated glycerol triester (OGT), providing greater relief for benefit for xerostomia symptoms by lubricating and protecting the tooth enamel and oral mucosa.^1,6^ OGT is a lipid capable of adhering to and protecting the oral mucosa and it yields better results when compared to aqueous electrolyte sprays.^6^ Some formulations may contain electrolytes and antimicrobial agents to buffer pH, inhibit biofilm accumulation, and protect dental enamel hydroxyapatite from demineralization. The presence of weak acids can stimulate saliva secretion from parotid and sublingual glands.^7-9^ Other substances, such as tocopherol and Aloe vera, have anti-inflammatory and healing properties.^10-12^ Although the therapeutic benefit of urea against radiotherapy-induced xerostomia has not yet been reported in the literature, it holds significant potential. Commonly indicated for the prevention of radiation-induced dermatitis,^13^ urea at low concentrations (2%–10%) is a potent moisturizing agent, regulating transepithelial water loss and enhancing the ability of the epithelium to attract and maintain hydration in cases of xerosis.^13,14^ Currently, there is some evidence supporting the use of urea mouthwash for the relief of xerostomia symptoms in burning mouth syndrome^15^ and its potential role in anticaries^10,16^and antimicrobial protection.^14^

The aim of this study was to evaluate the effectiveness of an over-the-counter salivary substitute and a compounded mouthwash containing urea in relieving symptoms of radiotherapy-induced xerostomia through a double-blind randomized controlled clinical trial.

## Methods

### Study design and eligibility criteria

This parallel and double-blind randomized clinical trial followed the CONSORT statement and was approved by the Institutional Research Ethics Committee (Plataforma Brasil https://plataformabrasil.saude.gov.br/, CAAE: 13451019.6.0000.0075) and registered in the Brazilian Registry of Clinical Trials https://ensaiosclinicos.gov.br/, Register: RBR-5q6h4qb (Universal Trial Number: U1111-1260-0509).

The enrolled patients were selected from those who received dental treatment at the Interdisciplinary League of Oral Neoplasms at the School of Dentistry of the Universidade de São Paulo (LINB-FOUSP) between January and June of 2020. All these patients had been previously diagnosed and treated for head and neck cancer, at least one year before their enrolment in this trial. Their dental treatment was performed on average 3 months before inclusion and consisted of oral hygiene guidance, periodontal scaling, caries removal and, in some cases, endodontic treatment followed by resin composite restorations and prosthetic rehabilitation. Extractions were less common, but performed under antibiotic therapy, with minimal trauma and occlusive suturing. No cases of osteoradionecrosis were observed until complete healing.

Upon enrollment, the participants were considered to have good oral health and were free from additional complications associated with radiotherapy, such as mucositis, candidiasis, and radiation-related caries. All patients agreed to their participation and signed an informed consent form. The participants were included according to the criteria for xerostomia symptoms and previous radiotherapy for oral or pharyngeal cancer treatment. None of the participants had previously received interventions to prevent xerostomia or hyposalivation during head and neck radiotherapy. The exclusion criteria included individuals with a history of hypersensitivity to the mouthwash formulations or those who refused to participate. Screening and selection of the participants were conducted by three trained and calibrated operators.

### Study protocol

The included patients were randomly allocated to the Xerolacer and urea 10% solution groups according to the sequence generated by the Random Allocation v.1.0.0 Software (M. Saghaei, Isfahan University of Medical Sciences – Isfahan, Iran). The sequence generation and preparation of the mouthwash solutions were carried out by a researcher who was not involved in patient management, data collection, or data analysis.

The initial assessment of xerostomia symptoms and the data collection during the study were carried out by another researcher, who received the mouthwash bottles containing 500 mL of Xerolacer (LACER S.A.U. – Barcelona, Spain) or 500 mL of a compounded 10% urea mouthwash with tutti-frutti flavoring to be handed out to the research participants. The formulation of both mouthwashes is described in Table 1. Each participant received two mouthwash bottles to ensure sufficient supply for mouth rinsing throughout the study. The bottles were white, opaque, and unlabeled, numbered only with the randomized inclusion sequence, thus blinding both researcher and patient to the study protocol.

**Table 1:** Xerolacer® and Urea mouthwashes formulations listed by manufacturer and related function.

Variables	Composition	Function
Xerolacer*	Sodium monofluorophosphate and Sodium fluoride (1,400 ppm)	electrolyte (1)
	Potassium glycyrrhizate	soothing agent (2)
	Vitamin E acetate (tocopheryl acetate)	antioxidant (3)
	Excipient for xerostomia based on mineral salts and lubricants:	
	Aqua	solvent
	Hydrogenated starch hydrolysate	humectant (4)
	Xylitol	sweetener (1)
	Propylene glycol	humectant (4)
	Glycerin	humectant (4)
	Hydroxyethylcellulose	emulsifier (5)
	PEG-40 hydrogenated castor oil	surfactant (4 and 6)
	Hydroxyacetophenon	antioxidant (2 and 3)
	Panthenol	humectant (4)
	Disodium EDTA	chelating agent (7)
	Aroma	flavoring agent
	O-Cymen-5-ol	antimicrobial action (8)
	Aloe barbadensis leaf juice	humectant (2 and 4)
	Sodium chloride	electrolyte (9)
	Potassium chloride	electrolyte (9)
	Sodium hydroxide	electrolyte (9)
	Citric acid	weak acid (9 and 10)
	Malic acid	weak acid (10)
	Sodium citrate	chelating agent (8)
	Potassium phosphate	electrolyte (9)
Urea	Urea (concentration: 10%)	humectant (4)
	Aroma (tutti-frutti)	flavoring agent
	Purified water	solvent

*Source: https://laceroralhealth.com/productos/xerolacer-colutorio/

(1) prevention of dental caries; (2) prevention of mucosal irritation; (3) facilitate epithelial regeneration; (4) moisturizing agent; (5) mucoadhesive film former; (6) foaming properties; (7) preservative; (8) reduction of dental plaque buildup; (9) buffering agent, maintenance of pH balance; (10) stimulation of salivary secretion.

The participants were instructed to rinse 10 mL of the undiluted solution thoroughly in the oral cavity for one minute, followed by expectoration. This procedure was repeated three times a day after toothbrushing for a period of 30 consecutive days.

### Data collection and analysis

The included patients were assessed at the time of inclusion (D1) and re-evaluated upon completion of the study protocol (D30) to determine the severity of dry mouth sensation. The numeric rating scale (NRS) and the Brazilian-Portuguese versions of the Summated Xerostomia Inventory-5 (SXI)^17^ and Oral Health Impact Profile-short form (OHIP-14) were used for the assessments and reassessments.^18^ The NRS and both questionnaires were administered through interviews conducted by the researcher, who was unaware of the participants’ group allocation. All participants were advised to ask the interviewer to repeat the question or provide further clarification if they did not understand a question, before giving an answer.

The demographic characteristics of the participants included in both groups were compared using Fisher’s exact test and the unpaired t-test. The NRS data and the questionnaires before and after the therapy in both groups were analyzed using repeated-measures two-way ANOVA and the Šidák post-hoc tests, all conducted using GraphPad Prism v.9.0.1 (GraphPad Software – Boston, USA).

## Results

**Figure 1:**
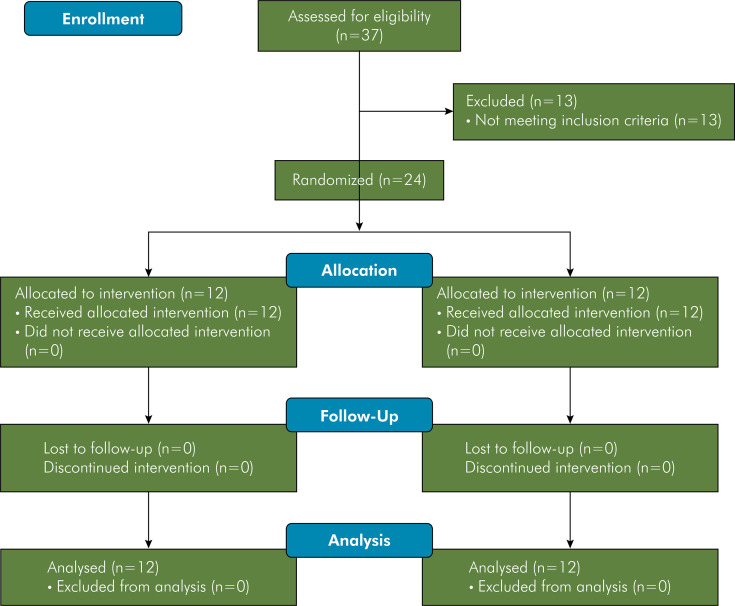
CONSORT flowchart showing clinical trial screening, eligibility, and inclusion.

**Figure 2:**
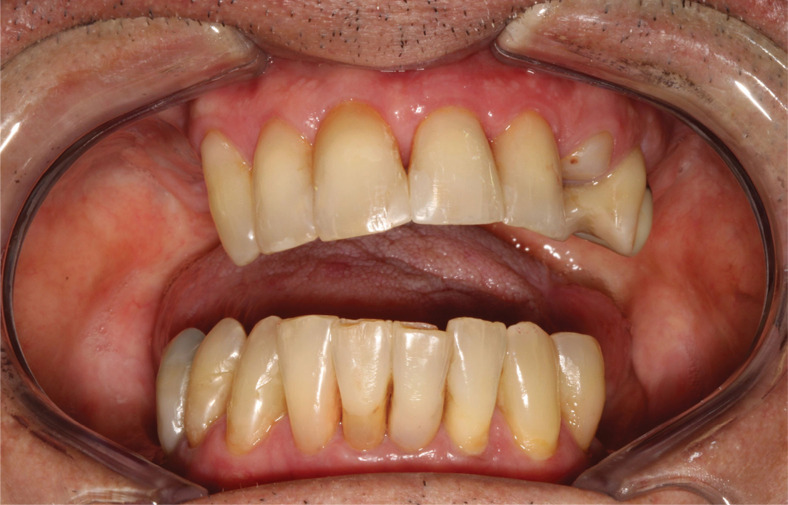
Clinical image of a participant who underwent surgery and radiotherapy for squamous cell carcinoma of the tongue. Presence of symptoms and signs of radiation-induced xerostomia, including thick and frothy saliva, shortening of tongue papillae, glassy appearance of the gingival mucosa, and teeth with cervical resin composite restorations due to radiation-induced caries.

A total of 24 participants who met the eligibility criteria were included in the study (Figure 1). Of these, 23 underwent conformal radiotherapy (3D-RT) involving the head and neck region, with a total radiation dose ranging from 46 to 76 Gy, leading to significant and permanent salivary flow impairment. Only one participant, diagnosed with nasopharyngeal squamous cell carcinoma, was treated with intensity-modulated radiation therapy, having received a total dose of 70 Gy. Clinically, all the participants, in addition to reporting discomfort due to oral dryness, presented some features indicative of severe xerostomia, such as frothy saliva, generalized shortening of tongue papillae, glassy appearance of the oral mucosa, and adherence of the mirror to the oral mucosa during clinical examination (Figure 2). Other complications caused by radiotherapy, such as candidiasis and radiation-related caries, were treated before inclusion in this trial. Other demographic and cancer diagnosis and treatment-related data are presented in Table 2.

**Table 2:** Demographic and oncological characterization of the oral cavity and pharyngeal cancer participants included in both study groups.

Variables	Xerolacer	Urea	p-value
		10% solution	
	(n = 12)	(n = 12)	
Sex			1.00*
Male	7	6	
Female	5	6	
Age			0.76**
Mean	60.3	59.3	
Variation (min–max)	48–74	42–72	
Smoking			1.00*
Yes	9	9	
No	3	3	
Diagnosis			0.47*
Squamous cell carcinoma	12	10	
Multiple myeloma	0	2	
Tumor site			
Oral cavity			
Lip	0	1	
Tongue	4	1	
Floor of the mouth	2	2	
Mandible	1	2	
Pharynx			
Oropharynx	3	5	
Nasopharynx	0	1	
Cervical lymph node	2	0	
Cancer treatment			0.31*
Surgical resection	8	11	0.66*
Neck dissection	9	7	1.00*
Adjuvant chemotherapy	4	3	
Radiotherapy - total dose (Gy)			
Mean	66.7	61.7	0.37**
Variation (min–max)	56–76	46–72	
Radiotherapy - time of conclusion (months)			1.00*
< 12	0	0	
12–36	7	8	
> 36	5	4	

*Fisher’s exact test, ^**^t-test for independent samples.

Column = mean, error bar = standard error, statistical analyses = *p < 0.05, ** p< 0.01, ***p < 0.001.

The Xerolacer and urea 10% solution groups exhibited homogeneous distribution in terms of sex, age, cancer diagnosis, tumor site, and cancer treatment, including the total dose and time of conclusion of head and neck radiotherapy (p > 0.05).

**Figure 3:**
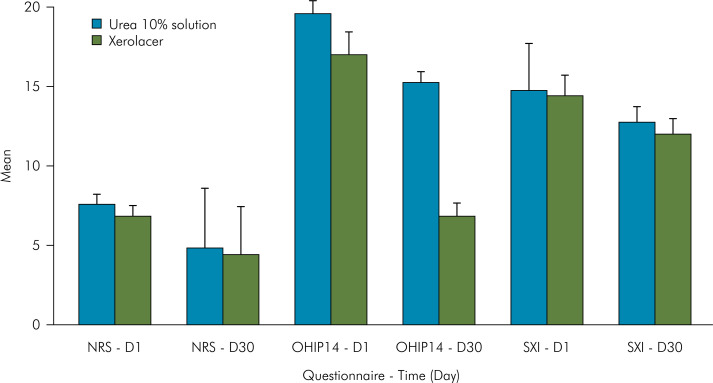
Assessment of xerostomia symptoms through the numerical rating scale, the Oral Health Impact Profile-short form, and the Summated Xerostomia Inventory-5 before and after 30 days of using the over-the-counter (Xerolacer) and compounded (Urea 10% solution) mouthwashes.

Both groups showed a significant reduction in overall xerostomia symptoms, as assessed through the NRS, after 30 days of mouthwash use (p < 0.01, d = 0.82), with an average symptom reduction of 35% in the Xerolacer group and 40% in the urea 10% solution group (Figure 3), with no statistically significant differences between the groups. A similar result was obtained using the SXI tool (p < 0.05, d = 0.74), with a significant reduction in the severity of xerostomia symptoms (Figure 3).

Only the Xerolacer group exhibited a statistically significant reduction in the OHIP-14 score (p < 0.01, d = 1.18), with an average decrease of 54%, reflecting a reduction in the impact on oral symptom-related quality of life (Figure 3).

## Discussion

This study assessed the effectiveness of the Xerolacer and urea 10% solution mouthwashes in relieving radiotherapy-induced xerostomia. The Xerolacer formulation contains humectants, antioxidants, antimicrobial agents, electrolytes, and weak acids. Together, these components have moisturizing properties that help prevent mucosal irritation, maintain pH balance, and stimulate salivary secretion, thereby preventing dental caries. The rationale for the use of urea 10% solution is based on its moisturizing properties,^13,14^ and both solutions have been previously associated with symptom relief in drug-induced xerostomia^11^ and xerostomia caused by burning mouth syndrome.^15^

The general characteristics of the study sample are consistent with epidemiological estimates of head and neck cancer,^19^ with most participants diagnosed with squamous cell carcinoma (91.6%) and smoking habit (75%), with a mean age of around 60 years. The most common sites for the lesions were the oropharynx (33.3%), tongue (20.8%), and floor of the mouth (16.6%).

It is estimated that doses of ionizing radiation exceeding 20 Gy, targeting the parotid gland, can lead to a significant reduction in salivary flow. While partial recovery of stimulated salivary flow may be observed after 18 months of treatment, this sequela continues to have a negative impact on the quality of life of these individuals.^20^ The substantial impact of this complaint on the participants included in both groups of our study can be explained by the high radiation dose (greater than 60 Gy) to which they were exposed.

This impact represents a significant challenge in the management of these individuals, and efforts to develop strategies to mitigate radiotherapy toxicity are necessary. Some alternatives, such as adopting cancer treatment approaches with organ preservation, i.e., intensity-modulated radiation therapy, are recommended. The sample consisted predominantly of individuals who received 3D-RT; therefore, a higher degree of xerostomia severity was expected due to the broader reach of this modality of head and neck radiotherapy, which includes healthy tissues such as the parotid glands.^20^ Various factors contribute to disparities in the selection of newer or more conservative radiation therapy delivery techniques, including age, race, and socioeconomic status.^21^ The participants included in this trial came from Brazilian public health services that did not always have IMRT technology available at the time of their diagnosis and treatment.

Only one participant in our trial was treated with IMRT due to a diagnosis of nasopharyngeal squamous cell carcinoma. Nasopharyngeal neoplasms, however, require high radiation doses to both parotid glands, even with IMRT technology, ultimately resulting in poor overall outcomes in terms of toxicity.^22^

Saliva substitutes, whether in the form of sprays, liquids, or gels, are intended to provide a moisture-retaining coating over the oral mucosa. In general, these products offer a palliative and limited effect for xerostomia and should be applied frequently throughout the day. The Cochrane Collaboration systematic review^6^ on the effectiveness of various saliva substitutes concluded that formulations containing OGT are more effective than aqueous electrolyte formulations in controlling dry mouth symptoms, but strong evidence to recommend these therapies is still lacking. The Multinational Association of Supportive Care in Cancer/International Society of Oral Oncology (MASCC/ISOO) guideline^5^ recommends topical mucosal lubricants or saliva substitutes for relieving xerostomia symptoms in cancer patients, reinforcing the brief duration of these effects.

Although the products studied in this trial have shown promise in relieving xerostomia symptoms, additional research is warranted, particularly concerning complex formulations such as Xerolacer. A recent systematic review that focused on 1% malic acid, a minor component of Xerolacer, demonstrated its sialogogue effect in managing xerostomia, highlighting the importance of combining malic acid with xylitol and fluorides to counteract the pH reduction that it induces.^23^

A low concentration of urea solution (around 10%) serves as a potent moisturizer and facilitates the penetration of other molecules through the skin, enhancing the effects of drugs used in combination therapies, such as antifungals.^14^ Additionally, in vitro evidence suggests that urea solution may contribute to anticaries protection.^10,16^These potential properties could benefit patients experiencing radiotherapy-induced xerostomia, who face a heightened risk of caries and opportunistic infections due to this side effect of cancer treatment. The present study demonstrated similar efficacy of both tested mouthwashes in alleviating xerostomia symptoms, supporting the possible use of a 10% urea solution in cases in which over-the-counter products are unavailable or prohibitively expensive. However, specific clinical studies are necessary to elucidate anticaries protection and other potential benefits provided by urea.

Considering the high bias observed in published studies, it is recommended that studies in this field assess whether the treatments are palatable, effective in reducing xerostomia, as well as their effects on the quality of life of those with chronic dry mouth symptoms.^6^ Therefore, the evaluation of the effectiveness of these products focuses on patient complaints about the subjective severity of dry mouth symptoms. Thus, appropriate patient-reported outcome measures (PROMs) should be assessed in order to achieve clinically meaningful results.^24^ For our trial, we selected three PROMs (NRS, SXI, and OHIP-14) in an attempt to properly measure symptoms, functional status, and health–related quality of life, respectively. The results obtained with the NRS and SXI tools were consistent and presented respectively high and moderate effect sizes, reflecting similar results of both mouthwashes for the relief of xerostomia symptoms. Conversely, oral health-related quality of life (OHIP-14) was significantly improved, with a high effect size, only in the Xerolacer group.

The OHIP-14 can be considered a general PROM that analyzes the influence of oral health on quality of life through different domains, including functional limitation, physical pain, psychological discomfort, physical disability, psychological disability, social disability, and social disadvantage. Condition–specific PROM, such as the NRS and SXI, are argued to have greater validity and responsiveness to changes in the participant’s condition.^24^ Additionally, from the investigators’ perspective, participants found them easier to answer. On the other hand, the OHIP-14 assessed aspects related to psychological and social disability, which required participants to exert more effort and take additional time to reflect on their own condition and the challenges in their personal and social relationships, leading to difficulty in answering the questions. Application of the OHIP-14 revealed an observed statistical difference between both mouthwashes, which should be interpreted with caution because of the small sample size. Nevertheless, it underscores the need to combine general and condition-specific PROMs in the evaluation of radiation-induced xerostomia symptoms.

Finally, all participants reported good tolerability and palatability in both evaluated groups and expressed a desire to continue using the products even after the research period, receiving prescriptions and remaining under dental care follow-up.

This study has some limitations, such as the small sample size and the absence of a consensus on relevant PROMs for radiotherapy-induced xerostomia. A recently published study investigating the use of pilocarpine spray,^25^ a salivary stimulant, in the management of radiotherapy-induced xerostomia, used the same PROM tools as in our study. However, another study that assessed various PROMs for radiotherapy-induced xerostomia^26^ did not consider the OHIP-14 to be a relevant tool for this assessment and also pointed out deficiencies in measurement properties and methodological quality of the xerostomia inventory, from which the SXI is derived. Determining the most suitable tools for this assessment is of paramount importance for the planning of future studies. Moreover, additional studies are required to evaluate the sustainability of the effect over an extended follow-up period, particularly regarding its impact on quality of life.

## Conclusion

Both mouthwashes can produce a transient beneficial effect on radiotherapy-induced xerostomia symptoms. The use of Xerolacer had a more pronounced impact on enhancing the oral health-related quality of life in a 30-day period.
